# Parallel expression evolution of oxidative stress-related genes in fiber from wild and domesticated diploid and polyploid cotton (*Gossypium*)

**DOI:** 10.1186/1471-2164-10-378

**Published:** 2009-08-17

**Authors:** Bhupendra Chaudhary, Ran Hovav, Lex Flagel, Ron Mittler, Jonathan F Wendel

**Affiliations:** 1 Department of Ecology, Evolution and Organismal Biology, Iowa State University, Ames, Iowa 50011, USA; 2 School of Biotechnology, Gautam Budhha University, Greater Noida, 201 308 UP, India; 3 Department of Biochemistry and Molecular Biology, MS200, University of Nevada, Reno NV 89557, USA; 4 Department of Plant Sciences, Hebrew University of Jerusalem, Givat Ram, Jerusalem 91904, Israel

## Abstract

**Background:**

Reactive oxygen species (ROS) play a prominent role in signal transduction and cellular homeostasis in plants. However, imbalances between generation and elimination of ROS can give rise to oxidative stress in growing cells. Because ROS are important to cell growth, ROS modulation could be responsive to natural or human-mediated selection pressure in plants. To study the evolution of oxidative stress related genes in a single plant cell, we conducted comparative expression profiling analyses of the elongated seed trichomes ("fibers") of cotton (*Gossypium*), using a phylogenetic approach.

**Results:**

We measured expression changes during diploid progenitor species divergence, allopolyploid formation and parallel domestication of diploid and allopolyploid species, using a microarray platform that interrogates 42,429 unigenes. The distribution of differentially expressed genes in progenitor diploid species revealed significant up-regulation of ROS scavenging and potential signaling processes in domesticated *G. arboreum*. Similarly, in two independently domesticated allopolyploid species (*G. barbadense *and *G. hirsutum*) antioxidant genes were substantially up-regulated in comparison to antecedent wild forms. In contrast, analyses of three *wild *allopolyploid species indicate that genomic merger and ancient allopolyploid formation had no significant influences on regulation of ROS related genes. Remarkably, many of the ROS-related processes diagnosed as possible targets of selection were shared among diploid and allopolyploid cultigens, but involved different sets of antioxidant genes.

**Conclusion:**

Our data suggests that parallel human selection for enhanced fiber growth in several geographically widely dispersed species of domesticated cotton resulted in similar and overlapping metabolic transformations of the manner in which cellular redox levels have become modulated.

## Background

Reactive oxygen species (ROS), including singlet oxygen, superoxide anions, hydrogen peroxide (H_2_O_2_), and hydroxyl radicals, are generated by oxidative reactions and other metabolic processes in cells and could have deleterious effects on growth and survival [1-4]. Naturally, cells have evolved mechanisms to scavenge excess ROS to prevent cellular damage, including up-regulation of antioxidant defense mechanisms [5-8]. In addition to the necessity of controlling excess potentially damaging ROS, eukaryotes have harnessed ROS as signaling molecules for a diverse array of regulatory processes, including responses to abiotic and biotic stresses, regulation of growth and development, and control of programmed cell death [2-5,9-11]. Because of their important roles as signaling molecules, as well as their toxicity at higher levels, ROS concentrations are finely tuned and developmentally regulated by a complex gene network (at least 152 genes in *Arabidopsis*; [2]), which collectively control and modulate ROS metabolism [2,5].

Recently, several ROS-related signal transduction and sensing components have been identified, including kinases, calcium channel proteins and redox-sensitive transcription factors [12-17]. The role of ROS has also been studied in cell wall biosynthesis, where ROS have been shown to be involved in lignin biosynthesis, cross-linking reactions between cell wall components, and loosening of cell walls in growing tissues [18-22]. ROS have been proposed to be involved in regulating cell growth in root hairs and pollen tubes, for example, via NADPH oxidases that control development by generating ROS and regulate cell expansion through the activation of calcium channels [23]. The role of Ca^2+ ^flux in ROS signaling is also well-characterized and involves calcium binding proteins, such as calmodulin and NADPH oxidases [24,25]. Stimulation of a Ca^2+ ^influx into the cytoplasm through NADPH oxidase-derived ROS, and in turn, activation of NADPH oxidase to produce ROS also establish a positive feedback regulation maintaining growth in expanding root hair cells [26]. However, exogenous application of H_2_O_2 _attenuated the rate of root hair growth with a prolonged rise in Ca^2+ ^after inhibition of growth [27]. ROS may also alter cell-wall properties and participate in their metabolism, as shown *in vivo *in radish seeds and maize roots [19,28]. In young cotton "fibers", which are single-celled epidermal trichomes, H_2_O_2 _appears to be important for the differentiation of the cellulose-rich secondary cell wall, and H_2_O_2_levels are finely regulated [20,29]. Additionally, exogenous H_2_O_2 _levels are regulated by redox status-related antioxidant enzymes including Cu/Zn-superoxide dismutase (CSD) that localizes to secondary cell walls of developing cotton fibers and is involved in cell wall growth [22]. Redox levels in cotton fiber cells are important for stability of cellulose synthases, necessary for cellulose biosynthesis during fiber elongation and secondary wall formation [30].

**Figure 1:**
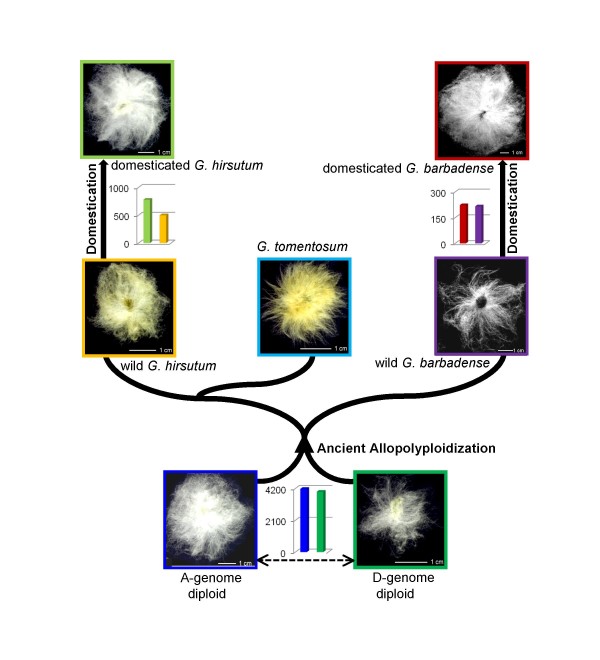
**Evolutionary history of diploid and allotetraploid cotton (*Gossypium*)**. Phylogeny of the genus is shown, with the history of repeated domestication at both the diploid (n = 13) and polyploid (n = 26) levels. The ancestral A- and D-genome diploids are inferred to have diverged from a common ancestor ~5–10 million years ago, prior to genomic merger in a common polyploid nucleus ~1–2 million years ago [32]. The newly evolved allopolyploid lineage subsequently diversified into five species (three used for microarray analysis are shown). Two allotetraploid species, *G. hirsutum *(source of 'upland cotton') and *G. barbadense *(source of 'Pima cotton'), and the diploid species *G. arboreum *were domesticated by humans within the past ~7000 years [35,36]. In the present study, we used models of the two progenitor diploids, *G. arboreum *(A-genome) and *G. raimondii *(D-genome), and both wild and domesticated forms of *G. hirsutum *and *G. barbadense*. We also included *G. tomentosum*, a wild allopolyploid from Hawaii. A representative image of a single seed at maturation is also shown for each species, with attached trichomes ("cotton fiber") with average fiber length (*G. raimondii *= 1.6 cm; *G. arboreum *= 3.0 cm; wild *G. hirsutum *= 2.0 cm; domesticated *G. hirsutum *= 3.9 cm; wild *G. barbadense *= 2.4 cm; domesticated *G. barbadense *= 4.3 cm; *G. tomentosum *= 1.2 cm) (modified from Applequist *et al*. [31]. Arrows denote microarray comparisons between species, with graphs designating the number of differentially up-regulated genes (*P *< 0.05 and FDR < 0.01) in each species, using the same color codes as in the seed image outlines.

Since ROS levels are important to cell growth, the possibility exists that ROS modulation has been responsive to selection pressure. The genus *Gossypium *is an excellent model for studying the evolution of ROS modulation because cotton fiber represents a highly derived modification of epidermal seed trichomes. These trichomes have the experimental advantage of being single-celled and readily detached for *in vitro *studies of gene expression or for other purposes. From an evolutionary standpoint, trichome length, color and form vary considerably among the ~50 mostly wild species in the genus [31,32], thus providing a natural system in which to investigate the evolutionary relationships between cell growth and ROS modulation. Finally, *Gossypium *includes both diploids and allopolyploids, of which four species (two diploid and two allopolyploid) were independently domesticated for use as feed and fiber crops [32] (Figure 1). This evolutionary context, and especially the multiple, parallel replicated "experiments" of domestication over the past ~7000 years, provide a unique opportunity to explore the connections between cell growth, natural and human-mediated selection, and ROS modulation.

Initial insight into these relationships has emerged from recent comparative gene expression profiling experiments. In a comparison between a short-fibered, wild diploid species (*G. longicalyx*) and a domesticated diploid cotton (*G. herbaceum*), Hovav *et al*. [33] showed that during fiber development several stress responsive genes were up-regulated in the wild species, whereas ROS scavenging and signaling genes were comparatively over-expressed at the same developmental stages in the domesticated species. Analogous results were obtained in another microarray comparison in cotton, this time between wild and domesticated forms of the allopolyploid species *G. barbadense*. In this latter study, a greater number of antioxidant and ROS signaling genes were up-regulated in the domesticated accession early in fiber development, whereas at the same developmental stages genes associated with ROS production were over-expressed in the wild accession [34].

Here we build upon these earlier observations with new analyses and experiments to explore the expression of ROS-related genes during various evolutionary stages encompassing several orders of magnitude of differences in time, including ~5–10 millions of years of diploid species divergence, the diversification of wild allopolyploid species over the past 1–2 million years, and two parallel domestications of allopolyploid species approximately 5000 years ago [35,36]. We use high-resolution microarray methodology [37] to comparatively study gene expression for ~42,000 genes in diploid and allotetraploid cotton species at an early stage of fiber development. By restricting our attention to ROS-related processes, we hoped to shed light on how ROS gene expression may have been altered in this single cell type during diploid divergence and natural allopolyploid formation, and by human selection pressure associated with repeated domestication.

## Results and discussion

### ROS-related gene expression evolution at the diploid level

To explore the evolution of oxidative stress related genes during diploid divergence, we assayed mRNA expression levels in fiber cells from domesticated *G. arboreum *and from wild *G. raimondii *at 2 days post-anthesis (hereafter "dpa"). This comparison encompasses natural species evolution in addition to human-mediated selection associated with domestication of the former species, which has spinnable fibers that are several-fold longer than the short, tightly adherent, commercially useless fibers of the former species (Figure 1). Our rationale for exploring fibers from 2 dpa was based on our earlier observations that oxidant and antioxidant genes are up- or down-regulated early in fiber development [33,34], but not later. For example, among the highest 100 differentially expressed genes in a wild accession of *G. barbadense *at 2 dpa, more than a third are potentially involved in stress-related processes; at later stages of fiber development, there were few such genes [34]. Using a custom microarray, this analysis revealed a high level of differential expression, using relatively stringent statistical criteria. Genes were considered significant if the *q *values, adjusted for multiple testing [38], were < 0.01 (Figure 1). Approximately 20% of all genes were differentially expressed between the two species, with a relatively even distribution in terms of direction; that is, 4,175 genes (~10% of the genes on the array) were up-regulated in *G. arboreum*, whereas 3,984 genes (~9.5%) were up-regulated in *G. raimondii*. This observation of extensive expression evolution between homologous cells of congeneric species, on a 5–10 million year timescale, is consistent with other recent comparative expression profiling experiments [33], where ~22% of all genes were differentially expressed in 5 dpa fibers of *G. longicalyx *and *G. herbaceum *species, consistent with the closer phylogenetic relationship of these taxa [32,39].

Among these more than 8,000 differentially expressed genes, gene lists were filtered for biological processes corresponding to putative ROS producing, scavenging and cell-signaling mechanisms based on GO annotations, as reviewed in [1] and [2] (Table 1), yielding a total of 548 genes (251 and 297 up-regulated in *G. arboreum *and *G. raimondii*, respectively; for gene list see Additional file 1). Notably, up-regulated genes were asymmetrically distributed between the two species with respect to classes of ROS genes. Many genes with antioxidant and transferase activity, and potential ROS-mediated cell-signaling processes are up-regulated in *G. arboreum *fibers, including 'peroxidase activity', 'glutathione transferase activity' and 'epoxide hydrolase activity', and in contrast few genes were categorized as contributing to ROS production. In contrast, *G. raimondii *fibers had higher levels of expression for genes involved in ROS production, including 'glycolate oxidase activity' and 'H_2_O_2 _biosynthetic process', but lower levels of expression for genes involved in ROS scavenging (Tables 1 & Additional file 2).

**Table 1:** Functional categories based on significantly up-regulated genes associated with ROS metabolism in 2 dpa fibers from the domesticated diploid *G. arboreum *and wild diploid *G. raimondii *(*p *< 0.05 and FDR < 0.01).

Mechanism	Taxon with significantly greater expression	Functional category^*a*^	*Corresponding key genes*^*b*^
ROS production	*G. arboreum* *G. raimondii*	Photorespiration Glycolate oxidase activity NADPH:quinone reductase activity O_2_^-^/H_2_O_2 _production process Long-chain-fatty-acid-CoA ligase activity	*GOX, HPR* *GOX, HAO* *QR-like, SDR* *NADPH Oxidase* *ACX6*

ROS scavenging	*G. arboreum* *G. raimondii*	Peroxidase activity Antioxidant activity Glutathione transferase activity Ascorbate peroxidase activity Xanthophyll biosynthetic process Response to toxin Cellular response to stress Response to oxidative stress Epoxide hydrolase activity ----	*PRX, APX, GST* *PRX, GST, Cu/Zn SOD* *GSTs* *APX, APX (chl)* *ZEP* *GSTs* *GST, NTL* *PRX, APX, GST, HSPIII* *EPH*

Cell signaling	*G. arboreum* *G. raimondii*	Protein kinase regulator activity *ent*-kaurene oxidase activity Carbonic anhydrase activity Calcium ion signaling Phosphoglycolate phosphatase activity Protein kinase activity Response to salicylic acid stimulus Response to jasmonic acid stimulus Response to ethylene stimulus	*Cyclin D, CDK* *CYP450, GAs* *CAs* *CaDK, CaATPase, CBP* *PGP, HDL* *Ser/ThrPK, MAPK4* *MAPK, WRKY78, Myb1* *LOX, MAPKs, Myb* *MYBs, NADPHO, ERF*

^*a*^Blast2GO http://www.blast2go.de was used as a tool for Gene Ontology functional classification of sequences.

^*b*^Key ROS related genes are listed with their corresponding processes (a complete list can be viewed as supplementary material; Additional file 1). Abbreviations: *GOX *– Glycolate oxidase; *HPR *– hydroxypyruvate reductase; *HAO *– hydroxy-acid oxidase; *QR*-like – quinone reductase-like protein; *SDR *– Short-chain dehydrogenase/reductase; *ACX6 *– long chain acyl-CoA synthetase 6; *PRX *– Peroxidase; *APX*-Ascorbate peroxidase; *APX *(chl) – Thylakoid-bound ascorbate peroxidase; *GST *– Glutathione S-transferase; *SOD *– Superoxide dismutase; *ZEP *– zeaxanthin epoxidase; *NTL *– Nitrilase; *HSP *– Heat shock protein; *EPH *– Epoxide hydrolase; *CDK *– Cyclin dependent kinase; *CYP450 *– Cytochrome P450 monooxygenase; *GA *– Gibberellins; *CA *– Carbonic anhydrase; *CaDK *– Calcium-dependent protein kinase; *CaATPase*-calcium-transporting ATPase 1; *CBP *– Calmodulin-binding protein; *PGP *– Phosphoglycolate phosphatase; *HDL *– Hydrolase; *Ser*/*ThrPK *– Ser/Thr protein kinase; *MAPK4 *– Mitogen-activated protein kinase 4; *LOX *– Lipoxygenase1; *ERF *– Ethylene response factor 3a.

FDR = False discovery rate.

The foregoing results show parallels with earlier studies involving microarrays and cotton fiber development. Specifically, in comparisons between the diploid species *G. herbaceum *and *G. longicalyx*, as well as for wild vs. domesticated accessions of *G. barbadense *(Pima cotton), some genes involved with modulating H_2_O_2 _and other ROS levels were up-regulated in the accessions with longer fibers [33,34]. As noted above, inhibition of H_2_O_2 _production or application of exogenous H_2_O_2 _acts as a signal in cell wall differentiation in young cotton fibers, with extended cell elongation correlated with activation of antioxidant enzymes and premature formation of secondary walls being associated with increased H_2_O_2 _levels [20,29].

In addition to genes likely to be *directly *involved with ROS production and scavenging, many other genes may be stimulated through ROS signaling cascades that ultimately influence gene expression. For instance, although signaling molecules such as 'kinases' are known to be important in many cellular processes, some play roles in downstream signaling events associated with ROS. Serine/theronine protein kinases for example, play a major role in ROS sensing and the activation of MAPKs [40]. However, downstream signaling events associated with ROS sensing involve calcium and calcium binding proteins [24,25]. In addition, a number of transcription factors are important components of the oxidative stress response signal transduction network. These include heat shock transcription factors, Zn finger proteins and WRKY transcription factors [12-15]. To search for the involvement of these classes of genes less certain to be involved in ROS metabolism than those directly functioning in the production and scavenging of ROS, we conducted searches based on sequence similarity to angiosperm homologs (usually from *Arabidopsis*), and identified several genes up-regulated in both *G. arboreum *and *G. raimondii *that become candidates for having roles in ROS signaling (Table 1). These, however, require experimental characterization to substantiate a role in ROS signaling.

Modulation of ROS levels may also be linked to cell elongation, as indicated by the aforementioned work on hydrogen peroxide levels and fiber elongation [20,29]. As shown in Table 1, more processes involving antioxidant genes (ROS scavengers) are up-regulated in *G. arboreum *when compared to *G. raimondii *and *vice versa *for oxidant genes (ROS producing). Thus, *G. arboreum *fibers may experience less oxidative stress than *G. raimondii *fiber cells at the same developmental stage, suggesting a connection to the differences in fiber phenotype. This provokes the speculation that some of the stress-related biological processes and genes that are up-regulated in *G. arboreum *have achieved this through domestication.

**Figure 2:**
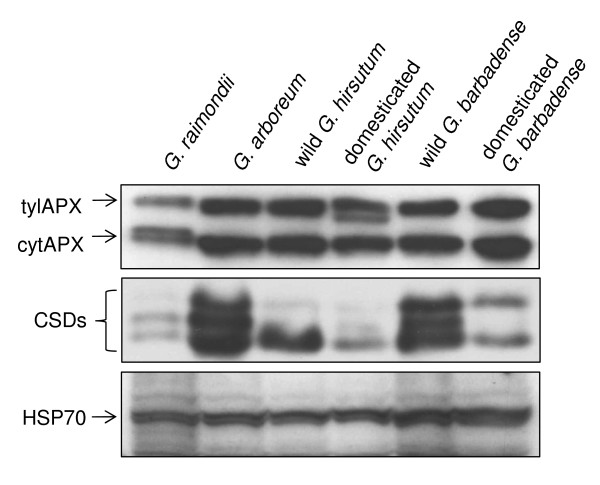
**Protein blot analysis from wild and domesticated diploid and polyploid species**. It is to show levels of thylakoid bound and cytosolic ascorbate peroxidase (tylAPX and cytAPX), Cu/Zn-Superoxide dismutase (CSD) and HSP70 proteins in 2 dpa fiber extracts. All experiments were repeated twice and representative results are shown.

As shown in Figure 2, up-regulated antioxidant genes in domesticated *G. arboreum *also show up-regulation at the protein level, validating our microarray interpretations. Protein blot analysis with thylakoid ascorbate peroxidase (tylAPX) and cytosolic APX (cytAPX), as well as Cu/Zn-superoxide dismutase (CSD), confirmed the up-regulation of some of these genes in *G. arboreum *in comparison to *G. raimondii *during diploid divergence and domestication (see Table 2 for comparable APX and CSD microarray results). In addition, co-expressed HSP70 genes in all six species also show equivalent expression at the protein level (Figure 2), providing additional support to the microarray results.

**Table 2:** Biological processes over-represented (*p *< 0.05) in domesticated *G. hirsutum *and domesticated *G. barbadense *relative to their wild forms.

Domesticated species	GO term	
	
	up-regulated	down-regulated
*G. hirsutum*	antioxidant activity (Chl-*APX*, *GST*, *CAT*, *TRX*)	regulation of O_2_^-^/H_2_O_2 _production process (NADPH oxidase)
	glutathione transferase activity (*GSTs*)	glycolate oxidase activity (*GOX*)
	Glycoside hydrolase activity(*GLH*)	cyclin-dependent protein kinase inhibitor activity
	peroxidase activity (*APX*, *GST*, *CAT*)	jasmonic acid and ethylene-dependent systemic resistance
	glutathione S-conjugate-exporting ATPase activity	
	removal of superoxide radicals (*TRX*)	
	oxidoreductase activity, acting on peroxides (*APX*, *GST*, *CAT*)	
	voltage-gated calcium channel activity (two pore Ca+ channels)	

*G. barbadense*	response to stress (*ESP*, *PRX, GH3*-like)	superoxide dismutase activity (Cu/Zn *SOD*)
	peroxidase activity (*PRX*)	carotenoid biosynthetic process (*GPS*)
	antioxidant activity (Chl-*APX, PRX*)	*ent*-kaurene oxidase activity (*CYP450*)
	regulation of epoxide hydrolase activity (*EPH*)	oxygen and reactive oxygen species metabolic process (*SOD*)
	voltage-gated calcium channel activity (two pore Ca+ channels)	photosystem II reaction center
	MAP kinase activity (*MAPK1*, *WIPK*)	
	receptor signaling protein serine/threonine kinase activity (*MAK*, *MAPKs*)	
	jasmonic acid mediated signaling pathway (*GH3*- -like)	
	glycolate oxidase activity (*GOX*)	

Key genes are shown in parentheses (a complete list can be viewed in Additional file 5).

Abbreviations: Chl-*APX *– Thylakoid-bound ascorbate peroxidase;, *GST *– Glutathione S-transferase;, *CAT *– Catalase, *TRX – *Thioredoxin reductase; *GLH *– Glycoside hydrolase; *APX *– Ascorbaste peroxidase; *PRX *– Peroxidase; *GOX *– Glycolate oxidase; *EPH *– Epoxide hydrolase; *MAPK *– Mitogen-activated protein kinase; *SOD *– Superoxide dismutase; *ESP *– Ethylene signaling protein; *GPS *– geranylgeranyl pyrophosphate synthase.

### Expression of ROS-related genes in wild allopolyploids

The observation that ROS-related genes are differentially expressed at the diploid level between the long- and short-fibered diploids *G. arboreum *and *G. raimondii *prompted us to test how differences were manifested in allopolyploids derived from the two genomes (A and D, respectively) represented by these two species. To explore this question for ROS-related genes in allopolyploid cotton fibers we utilized the phylogenetic framework represented in Figure 1 and microarray data from three of the five naturally occurring wild allopolyploids, i.e., *G. hirsutum, G. barbadense *and *G. tomentosum*, comparing mRNA abundances in these three species with 1:1 RNA mixtures of *G. arboreum *and *G. raimondii*, which represent a mid-parent value (MPV). We hypothesized that among allopolyploid species, domestication will have the strongest effect on ROS gene expression levels, whereas genome merger and doubling *per se *will have a weaker effect because there has not been human selection for increased fiber length; in this case our null hypothesis is that gene expression levels in wild allopolyploids should be, on average, approximately at the mean of the levels of the two progenitor diploids.

**Figure 3:**
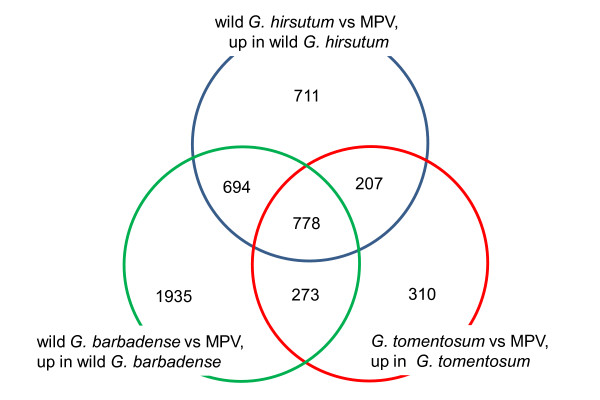
**Venn diagrams of gene expression evolution in allopolyploid cotton**. The numbers indicate genes classified as up-regulated in any wild polyploid species in comparison to the mid-parent values (MPV; estimated from a 1:1 RNA mix from the diploids *G. arboreum *and *G. raimondii*). Intersections denote genes shared by two or more of the three wild forms of the allopolyploids (wild *G. hirsutum*, wild *G. barbadense *and *G. tomentosum*).

Differences between the MPV and the three wild polyploid species (*G. hirsutum, G. barbadense *and *G. tomentosum*) led to varying numbers of differential expression in each case (Figure 3), with 778 shared genes that were up-regulated. These shared genes were thus inferred to represent the early effects of polyploidization, prior to speciation and later domestication. Categorization of these genes into biological processes revealed no significantly over-represented process related to ROS metabolism (see Additional file 3), consistent with our expectation that genome merger and polyploidization would not affect ROS-related genes in the wild species. We explored the data further for genes up-regulated in each of three wild species as well as genes shared by any two of the three species (numbers shown in intersections in Figure 3). No process related to ROS metabolism emerged for any single species, whereas for pairs of species, there was a marginally significant up-regulation of 'oxidases' in wild *G. hirsutum *vs wild *G. barbadense *as well as wild *G. barbadense *vs *G. tomentosum *(see Additional file 4).

### ROS-related genes and parallel domestication of cultivated allopolyploid cotton

We hypothesized that if ROS-related processes were sensitive to modification by selection via their putative relationship to cell elongation [33,34], then, and in contrast to the expectation for wild polyploids, domesticated varieties of the polyploid species would be expected to exhibit evidence of this at the gene expression level. Additionally, as noted above, two species of allopolyploid cotton, *G. hirsutum *and *G. barbadense *have been independently domesticated [32,35], thus allowing two tests of this hypothesis.

To test this we used the microarray data to perform wild/domesticated contrasts in both species. These analyses revealed 777 and 498 genes that were up- and down-regulated, respectively, in cultivated *G. hirsutum *versus its wild counterpart, and 223 and 216 that were up-and down-regulated, respectively, in cultivated *G. barbadense*, relative to its wild accession (Figure 1). Assessment of biological processes corresponding to these differentially up-regulated genes showed significant up-regulation of processes involving antioxidant genes in both domesticated species (see Additional file 2 & Table 2).

An important pattern emerged from these comparative expression data, namely, that shared processes between the two species were often observed but that they involved in different sets of genes. For example, the process 'peroxidase activity' in *G. hirsutum *includes ascorbate peroxidase, catalase and thioredoxin reductase genes, whereas in *G. barbadense *this process is represented by several peroxidase genes (see Additional file 2 &4). Similarly, different sets of genes in the 'antioxidant activity' category were up-regulated in the two species, i.e., catalase, glutathione S-transferases and thioredoxin in *G. hirsutum*, and peroxidases in *G. barbadense *(Table 2 & Additional file 5). These genes have been shown to function as antioxidants, assisting in the regulation of ROS levels and contributing to cell elongation under high redox conditions [8,29,41,42]. Our data suggest that parallel selection by humans operated on different genetic components of the fiber development program in *G. hirsutum *and *G. barbadense*, leading to parallelism at the metabolic level and in morphology.

In addition to parallel up-regulation of ROS-related processes, our data also reveal possible examples of parallel down-regulation. A case in point is for the process 'peroxide biosynthesis', which was down-regulated in the cultivated varieties of both *G. hirsutum *and *G. barbadense*, suggesting that down-regulation of ROS production accompanied domestication (Table 2). In addition to the foregoing examples of putative parallel responses to domestication, other ROS-related processes and genes were significantly differentially expressed in one of the two domesticated species. In domesticated *G. hirsutum*, the biological process 'cyclin-dependent protein kinase inhibitor activity', which harbors a gene for inhibiting serine/threonine kinase that has been shown to play crucial role in ROS sensing [40], was down-regulated. This observation raises the possibility that domestication entailed an repression of kinase inhibitor genes, enhancing the potential of a growing cell to sense toxic ROS levels and activate downstream processes to maintain optimal concentration. In domesticated *G. barbadense*, the processes 'superoxide dismutase' and 'carotenoid biosynthetic process' were down-regulated, both known to be important in ROS scavenging (Table 2) [6,41]. The microarray based interpretations were also confirmed with comparable results at protein level for Cu/Zn-superoxide dismutase (CSD) and tylAPX and cytAPX for wild and domesticated *G. hirsutum *and *G. barbadense *(Figure 2). Comparable to microarray data, higher levels of CSD is observed in wild *G. barbadense *in comparison to domesticated *G. barbadense *(Figure 2). However, although CSDs transcript levels in domesticated *G. hirsutum *are up-regulated in comparison to wild *G. hirsutum *(see Additional file 2), this is not observed in the protein blots (Figure 2). This inconsistency could be a result of antibodies cross-reacting with only some but not all of the CSD gene products, potentially not reacting with the specific CSD transcripts up-regulated in domesticated *G. hirsutum*, as well as differences in post-translational modification, or other factors.

### ROS-related genes and parallel domestication across ploidy levels and continents

**Figure 4:**
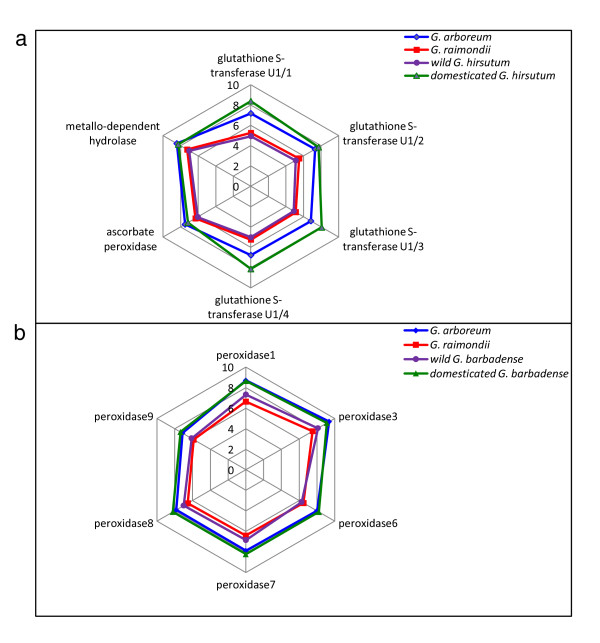
**Differential gene expression patterns for some ROS-related genes**. These genes are differentially up-regulated in *G. arboreum *and the domesticated polyploids in contrast to their relative wild relatives or ancestors. Each point on the polygons represents a gene and the scale corresponds to relative expression levels in the diploid model progenitor genomes (*G. arboreum *and *G. raimondii*) and the wild and domesticated forms of: (**a**) *G. hirsutum; *and (**b**) *G. barbadense*.

Because cotton domestication involved in two different allopolyploid species in the New World as well as two different diploid species in the Old World [32,36], a naturally replicated "experiment" of domestication provides the opportunity to evaluate potential parallelisms in ROS-related responses across ploidy levels and continents. To explore parallel recruitment of genes during domestication at the diploid and polyploid levels, we tabulated the differentially expressed genes (up-and down-regulated) that were *shared *between *G. arboreum *and domesticated *G. hirsutum *as well as between *G. arboreum *and domesticated *G. barbadense *(marked by asterisks in Additional file 2). A total of 178 and 38 shared genes were up-regulated and 102 and 22 were down-regulated in *G. arboreum *with *G. hirsutum *and *G. arboreum *with *G. barbadense *in comparisons to their wild relatives, respectively. Up-regulated genes shared by *G. arboreum *and *G. hirsutum *belong mainly to ROS scavenging classes, including glutathione S-transferase and ascorbate peroxidases (see Additional file 2), but also include putative ROS signaling genes such as serine/threonine kinase (see Additional file 5). Up-regulation in *G. arboreum *and *G. hirsutum *suggests that selection during domestication for agronomically more favorable fiber phenotypes resulted, in parallel, in the enhanced expression of similar genes. In contrast, *G. raimondii *and wild *G. hirsutum *have low levels of expression of these same genes, correlated with their much shorter fibers and temporally compressed development (Figure 4a) [31].

Shared up-regulated genes between *G. arboreum *and domesticated *G. barbadense *include a suite of peroxidase genes (see Additional file 2). These genes encode an important class of ROS-regulating enzymes, which eliminate toxic molecules generated as a byproduct of aerobic respiration [29]. As shown in Figure 4b, expression levels of peroxidase genes in domesticated *G. barbadense *were similar to those of *G. arboreum*, suggesting once again parallel selection under domestication at the diploid and polyploid levels.

## Conclusion

The present study implicates an oxidative stress responsive gene network as being involved in the evolution of elongated epidermal seed trichomes, providing the foundation for later human domestication of an important crop plant. We provide clues here into ROS related genes that may have been selected by humans, starting with initial domestication of wild perennial thousands of years ago, through the development of modern elite lines. Notably, the majority of up-regulated ROS-related processes are diagnosed as having become enhanced during domestication, at both the diploid and polyploid levels, as opposed to being a result of allopolyploid formation per se. This suggestion that expression evolution of oxidative stress related genes was primarily concomitant with domestication is bolstered by the remarkable observation that independent parallel domestication events, taking place in different hemispheres thousands of years ago under aboriginal human selection pressure, led to parallel recruitment of ROS scavenging and signaling genes in one diploid and two different polyploid species. Although this is true at the level of biological process, or perhaps metabolism, it is clear that the genesis of these similarities is only partially congruent at the genetic level. That is, different sets of antioxidant genes are up-regulated in the domesticates. This repeated metabolic transformation accompanying domestication would appear to be without precedent. An exciting prospect for future work will be to dissect this physiological parallelism into its responsible constituent genes, and to learn the mutational basis of their altered regulation or function. Notwithstanding our ignorance of the specific function of most of the genes implicated as important in this study, a generality emerges that the avoidance or delay of stress-like processes in domesticated species may play a large role in the enhance elongation of fibers in cultivated compared to wild forms, in conjunction with an increased ability to modulate cellular redox balance in the growing cell. It would be interesting to explore whether similar trends in the evolution of ROS gene expression accompanied the domestication of other crops and for other traits, for example tolerance to stressed environmental conditions or to enhanced growth of other organs.

## Methods

### Plant materials, RNA isolation and microarray preparation

Three biological replicates of seven *Gossypium *accessions were grown in the Pohl Conservatory at Iowa State University, Ames, IA: *G. arboreum*, *G. raimondii*, a wild form of *G. hirsutum *(accession TX2094), a domesticated form of *G. hirsutum *(accession TM1), a wild form of *G. barbadense *(accession K101), a domesticated form of *G. barbadense *(accession Pima S-7) and *G. tomentosum*. Because truly wild, as opposed to feral, forms of *G. barbadense *are difficult to verify, we selected the accession used based on earlier allozyme analysis (Percy and Wendel, 1990) and its relatively primitive morphology. These accessions include representatives of both diploid progenitor genomes ("A" and "D") of allopolyploid cotton [32](Figure 1), and both wild and domesticated forms of allopolyploid cotton (*G. hirsutum *and *G. barbadense*).

Flowers were tagged at anthesis and harvested at 2 days post-anthesis. For each of three biological replicates, ovules were excised, frozen in liquid nitrogen, and stored at -80°C. RNA extractions were performed following a modified hot borate procedure optimized for *Gossypium *[43]. From each pair of A_2 _and D_5 _replicates, an equimolar RNA mix (1:1 mix) was also generated to estimate mid-parent expression values (MPV). RNA samples were amplified with the TargetAmpTM 1-Round aRNA Amplification kit from Epicentre Biotechnologies (Madison, WI) and quantified and assessed for degradation using a Bioanalyzer (Agilent Technologies, Santa Clara, CA). All amplified RNA samples were sent to NimbleGen Systems (Madison, WI) for cDNA synthesis, labeling, and hybridization to custom microarrays developed from an assembly of cotton ESTs [37]. This custom microarray platform has the ability to measure homoeolog-specific gene expression for ~1500 genes in the allotetraploids, as well as overall gene expression for 42,429 unigenes.

### Microarray data processing and statistical analysis

Raw data values for each gene were natural-log transformed, median-centered, and quantile-normalized across all arrays. Following normalization, contig-level log ratio values were determined by calculating an average of log ratio values from 7 probes per gene for a total of 42,429 genes, using Tukey's biweight method [44,45]. A linear model as described [34], including effects for replication and genotype, was fitted to these contig-level data, allowing the estimation of all possible contrasts between *G. arboreum, G. raimondii*, their 1:1 mix, wild *G. hirsutum*, domesticated *G. hirsutum*, wild *G. barbadense*, domesticated *G. barbadense *and *G. tomentosum*. For each gene, differences were calculated using pair-wise contrasts between A_2 _vs D_5_, wild *G. hirsutum *vs 1:1 mix, wild *G. barbadense *vs 1:1 mix, *G. tomentosum *vs 1:1 mix, wild *G. hirsutum *vs domesticated *G. hirsutum *and wild *G. barbadense *vs domesticated *G. barbadense*. The 42,429 *p*-values from each comparison were converted to *q*-values using the method of [38]. These *q*-values were used to identify the number of differentially expressed genes for a given comparison when controlling for a false discovery rate (FDR) of 0.01. Blast2GO http://www.blast2go.de was used to infer biochemical pathways involved in a given comparison and to calculate statistical significance. The expression values for microarray experiments have been submitted to the database with following accession numbers: GSM432872; GSM432873; GSM432874; GSM432875; GSM432876; GSM432877; GSM432878; GSM432879; GSM432880; GSM432881; GSM432882; GSM432883; GSM432884; GSM432885; GSM432886; GSM432887; GSM432888; GSM432889.

### Validation of microarray results with massARRAY

To confirm the microarray-based interpretations, we used an application of a mass *ARRAY *based validation technique. The robustness of mass *ARRAY *technique has been demonstrated showing high correlation with RT-PCR [46]. We checked 18 randomly selected genes at 2 dpa in three biological replicates for homoeolog contributions to the transcriptome for domesticated *G. hirsutum *samples (for details of validation design, see Supplementary methods in Additional file 6). The mean value for each of three replicates was determined and compared with the estimates derived from the homoeolog-specific microarray. The correlation between SNP-specific microarray and mass *ARRAY*-based techniques is shown with high R^2 ^value (0.84) at *p*-value < 0.001 in Additional file 6.

### Protein blot analysis

Cotton fibers were mechanically separated from frozen ovular tissue using a modified liquid nitrogen and glass bead shearing procedure [47]. Total protein samples were extracted using a phenol-ammonium acetate/methanol method [48]. Briefly, ~250 mg of crushed fiber tissue was dissolved in extraction buffer (100 mM Tris-HCl, (pH 8.8), 10 mM EDTA, 900 mM sucrose, 0.4% 2-mercaptoethanol) at 4°C. The proteins were precipitated overnight with ammonium acetate/methanol solution at -20°C and the pellet, collected by centrifugation, was washed with ice-cold ammonium acetate/methanol (100 mM ammonium acetate in 100% methanol) and 80% acetone and dried. The total proteins pellets were solubilized into 1× Laemmli sample buffer (0.0625 M Tris-HCl (pH 6.8), 2% SDS, 25% glycerol, 5% 2-mercaptoethanol) and stored at -20°C until analysis. Protein gel blots were preformed as described [7]. Antibodies against cytosolic and chloroplastic APXs (ascorbate peroxidase) were obtained using a domain conserved to these proteins (a fragment of thylakoid APX from Lys^100 ^to Ile^341^) as described in [49]. Antibodies against Cu/Zn-superoxide dismutase (CSD) was obtained from Agrisera (Vannas, Sweden) and that against HSP70 was prepared as described by [50] using purified proteins as antigens. Primary rabbit antibodies were used in a 1:3000 dilution while secondary goat anti rabbit antibodies conjugated to horse radish peroxidase (Invitrogen, Carlsbad, CA) were used at a 1:20,000 dilution. ECL™ chemiluminescent detection (Amersham Biosciences, Buckinghamshire, UK) was performed as described in [7].

## Authors' contributions

BC and JFW designed the research work and wrote the manuscript. BC, RH and RM carried out the laboratory-based studies. BC and LF analysed the data. RM and JFW advised on numerous aspects of the study, reviewed the results and performed critical reading and editing of the manuscript. All authors read and approved the final manuscript.

## Supplementary Material

Additional file 1 **Biological processes with their corresponding genes up-regulated in *G. arboreum *in contrast to *G. raimondii***. The data provided represent the statistical comparison of two diploid species at early stage of fiber development. Click here for file

Additional file 2 **Gene annotation and significant over-representation of the ROS-scavenging network in cotton fibers (FDR < 0.01)**. The data provided represents all up- or down-regulated antioxidant genes in domesticated accession in comparison to their wild relatives. Click here for file

Additional file 3 **Common biological processes up- and down-regulated after polyploid formation in three wild allopolyploid species (wild G. hirsutum, G. tomentosum and wild G. barbadense) in contrast to the mid-parent value (MPV) from progenitor diploid genomes**. The data provided represents the up-regulated genes in all three wild species after polyploidization. Click here for file

Additional file 4 **Biological processes categorized based on differentially up-regulated unique genes in each of three wild polyploid species in contrast to the MPV, exclusive of any set of shared genes; and processes based on up-regulated genes shared by any two species (exclusive of up-regulated genes shared by all three wild species)**. The data provided represents the unique and shared biological processes observed to be up-regulated only in all three wild accessions studied. Click here for file

Additional file 5 **Assessment of biological processes for up-regulated genes in domesticated accessions relative to their wild forms**. The data provided represents all significant biological process observed to be up-regulated in two domesticated accession in comparison to their wild relatives. Click here for file

Additional file 6 **Validation of NimbleGen microarray values by mass *ARRAY*-based homoeolog-specific expression measurements. **(a) List of 18 homoeologous genes analyzed for validation of NimbleGen microarray values by mass *ARRAY*-based homoeolog-specific expression measurements. (b) Correlation between NimbleGen microarray data and mass *ARRAY*-based homoeolog-specific measurements. The relative expression of D-homoeolog detected at 2 dpa based on NimbleGen microarray signal information and plotted on the y-axis. The x-axis is the proportion of D-homoeolog that is observed based on mass *ARRAY *measurements. The best-fit trend line with R^2 ^correlation and *p*-value is shown on the graph for early stage of fiber development. The data provided represents the validation of homoeologous gene expression in the developing fibers from wild and domesticated accessions. Click here for file
