# Supplementary material for: Parallel expression evolution of oxidative stress-related genes in fiber from wild and domesticated diploid and polyploid cotton (Gossypium)
Source: BMC Genomics. 2009 Aug 17;10:378. doi: 10.1186/1471-2164-10-378 (PMC2907704; doi:10.1186/1471-2164-10-378)
Supplement: Additional file 3 — Common biological processes up- and down-regulated after polyploid formation in three wild allopolyploid species (wild G. hirsutum, G. tomentosum and wild G. barbadense) in contrast to the mid-parent value (MPV) from progenitor diploid genomes. The data provided represents the up-regulated genes in all three wild species after polyploidization. [file 1471-2164-10-378-S3.doc]

| **Additional file 3**. Common biological processes up- regulated after polyploid formation in three wild allopolyploid species (wild *G. hirsutum*, *G. tomentosum*, and *G. barbadense*) in contrast to the mid-parent value (MPV) from progenitor diploid genomes. | | | |
| --- | --- | --- | --- |
| **GO term** | **Biological process** | **FDR** | ***p*-value** |
| *Up-regulated in all three wild species*  GO:0044427 chromosomal part | | 1.37E-04 | 1.36E-07 |
| GO:0005694 | chromosome | 2.85E-04 | 5.66E-07 |
| GO:0000785 | chromatin | 2.87E-04 | 9.05E-07 |
| GO:0004163 | diphosphomevalonate decarboxylase activity | 3.25E-04 | 1.29E-06 |
| GO:0019287 | mevalonate pathway | 3.25E-04 | 1.29E-06 |
| GO:0006333 | chromatin assembly or disassembly | 5.64E-04 | 2.98E-06 |
| GO:0006325 | establishment and/or maintenance of chromatin | 6.44E-04 | 4.67E-06 |
| GO:0006323 | DNA packaging | 6.44E-04 | 4.67E-06 |
| GO:0009636 | response to toxin | 0.0011542 | 9.91E-06 |
| GO:0007001 | chromosome organization and biogenesis | 0.0011542 | 1.14E-05 |
| GO:0051276 | chromosome organization and biogenesis | 0.0011542 | 1.19E-05 |
| GO:0000786 | nucleosome | 0.00184537 | 1.86E-05 |
| GO:0031497 | chromatin assembly | 0.00440778 | 5.40E-05 |
| GO:0006334 | nucleosome assembly | 0.00440778 | 5.51E-05 |
| GO:0043228 | non-membrane-bound organelle | 0.013459 | 1.64E-04 |
| GO:0043232 | intracellular non-membrane-bound organelle | 0.013459 | 1.64E-04 |
| GO:0003777 | microtubule motor activity | 0.0160503 | 2.01E-04 |
| GO:0006259 | DNA metabolic process | 0.0177365 | 2.64E-04 |
| GO:0009404 | toxin metabolic process | 0.0201343 | 3.36E-04 |
| GO:0009407 | toxin catabolic process | 0.0201343 | 3.36E-04 |
| GO:0004033 | aldo-keto reductase activity | 0.0210678 | 3.49E-04 |
| GO:0005315 | inorganic phosphate transmembrane transporter activity | 0.0244086 | 3.54E-04 |
| GO:0065004 | protein-DNA complex assembly | 0.0250002 | 4.20E-04 |
| GO:0008146 | sulfotransferase activity | 0.0349159 | 6.51E-04 |
| GO:0005875 | microtubule associated complex | 0.0449751 | 7.82E-04 |
|  |  |  |  |
