# Supplementary material for: Parallel expression evolution of oxidative stress-related genes in fiber from wild and domesticated diploid and polyploid cotton (Gossypium)
Source: BMC Genomics. 2009 Aug 17;10:378. doi: 10.1186/1471-2164-10-378 (PMC2907704; doi:10.1186/1471-2164-10-378)
Supplement: Additional file 4 — Biological processes categorized based on differentially up-regulated unique genes in each of three wild polyploid species in contrast to the MPV, exclusive of any set of shared genes; and processes based on up-regulated genes shared by any two species (exclusive of up-regulated genes shared by all three wild species). The data provided represents the unique and shared biological processes observed to be up-regulated only in all three wild accessions studied. [file 1471-2164-10-378-S4.doc]

| **Additional file 4**. Biological processes categorized based on differentially up-regulated unique genes in each of three wild polyploid species in contrast to the MPV, exclusive of any set of shared genes; and processes based on up-regulated genes shared by any two species (exclusive of up-regulated genes shared by all three wild species). | | | | |
| --- | --- | --- | --- | --- |
| **Species** | **GO term** | **Biological process** | **FDR** | ***p*-value** |
| wild  *G. hirsutum* | GO:0005198 | structural molecule activity | 4.68E-08 | 6.97E-11 |
|  | GO:0009861 | jasmonic acid and ethylene-dependent systemic resistance | 0.001278 | 1.05E-05 |
|  | GO:0044249 | cellular biosynthetic process | 0.001517 | 1.52E-05 |
|  | GO:0045330 | aspartyl esterase activity | 0.022149 | 3.83E-04 |
| *G. tomentosum* | GO:0007568 | aging | 0.010456 | 2.83E-05 |
|  | GO:0019187 | beta-1,4-mannosyltransferase activity | 0.010456 | 3.93E-05 |
|  | GO:0051753 | mannan synthase activity | 0.011536 | 0.011469 |
| wild  *G. barbadense* | GO:0008173 | RNA methyltransferase activity | 7.30E-04 | 2.03E-05 |
|  | GO:0042286 | glutamate-1-semialdehyde 2,1-aminomutase activity | 0.025546 | 0.001368 |
|  | GO:0015431 | glutathione S-conjugate-exporting ATPase activity | 0.029719 | 0.001671 |
|  | GO:0006437 | tyrosyl-tRNA aminoacylation | 0.033386 | 0.001785 |
|  | GO:0006526 | arginine biosynthetic process | 0.041552 | 0.002381 |
|  |  |  |  |  |
| Common processes between wild *G. hirsutum* and *G. tomentosum* | | | | |
|  | GO:0016899 | oxidoreductase activity | 2.40E-04 | 7.37E-07 |
|  | GO:0008891 | glycolate oxidase activity | 2.40E-04 | 7.37E-07 |
|  | GO:0003677 | DNA binding | 0.009211 | 3.92E-05 |
|  | GO:0003973 | (S)-2-hydroxy-acid oxidase activity | 3.03E-06 | 2.27E-06 |
| Common processes between *G. tomentosum* and wild *G. barbadense* | | | | |
|  | GO:0030497 | fatty acid elongation | 0.039095 | 1.50E-04 |
|  | GO:0004239 | methionyl aminopeptidase activity | 0.039095 | 1.56E-04 |
|  | GO:0006397 | mRNA processing | 0.039095 | 2.77E-04 |
|  | GO:0004637 | phosphoribosylamine-glycine ligase activity | 0.041685 | 0.085737 |
| Common processes between wild *G. hirsutum* and wild *G. barbadense* | | | | |
|  | GO:0051276 | chromosome organization and biogenesis | 5.25E-09 | 0 |
|  | GO:0065004 | protein-DNA complex assembly | 5.25E-09 | 2.42E-11 |
|  | GO:0006259 | DNA metabolic process | 5.25E-09 | 3.04E-11 |
|  | GO:0003677 | DNA binding | 2.09E-07 | 5.73E-09 |
|  | GO:0003713 | transcription coactivator activity | 0.008385 | 2.48E-04 |
|  | GO:0003723 | RNA binding | 0.014022 | 5.04E-04 |
|  | GO:0051348 | negative regulation of transferase activity | 0.016699 | 5.55E-04 |
|  | GO:0010257 | NADH dehydrogenase complex assembly | 0.028703 | 0.001099 |
