# Supplementary material for: Parallel expression evolution of oxidative stress-related genes in fiber from wild and domesticated diploid and polyploid cotton (Gossypium)
Source: BMC Genomics. 2009 Aug 17;10:378. doi: 10.1186/1471-2164-10-378 (PMC2907704; doi:10.1186/1471-2164-10-378)

Supplementary Methods

NimbleGen microarray validation experimental design and methodology

Earlier in our laboratory, homoeolog-specific probes were created by first assembling EST contigs from A_2, D_5, and AD_1 libraries and then identifying homoeolog-specific SNPs within these contigs. These SNPs represent nucleotide differences between the A-and D-genome orthologs, and offer the possibility of diagnosing the genomic origin of transcripts in the allopolyploid nucleus. Using this strategy, we have designed and implemented a novel NimbleGen microarray platform capable of measuring homoeolog-specific expression in *Gossypium* species (Udall et al., 2006). This microarray features two classes of probes, ~35mer probe-pairs differing by an A- or D-genome homoeolog-specific SNP, and ~60mer generic probes (not specific to either homoeolog). Thus this microarray platform has the ability to measure expression from both the homoeologs, detected by the corresponding ~35mer homoeolog-specific probes, and total gene expression, detected by the ~60mer generic probes designed in areas of common sequence between both homoeologs.

RNA samples from cotton fibers at early developmental stage (2dpa) in three biological replicates have been analyzed on this NimbleGen microarray platform for gene expression. The mean values for randomly selected 18 homoeologous genes pairs from microarray based results were compared with the estimates derived from homoeolog-specific Sequenom mass spectrometry for validating the homoeolog-specific expression ratios (Supplementary Figure 1). Briefly, the mass*ARRAY* technology amplifies A- and D-derived cDNA transcripts in parallel, and then quantifies relative homoeolog abundance based on matrix-assisted laser desorption/ionization time-of-flight mass-spectrometry.

Supplementary Figure 1a. List of 18 homoeologous genes analyzed for validation of NimbleGen microarray values by massARRAY based homoeolog-specific expression measurements**.**

**Contig**	**SNP position**	**domesticated *G. hirsutum* 2 dpa Fiber**	
		NimbleGen %D expression	mass*ARRAY* %D expression
COTTON16_00001_452	1635	0.530	0.409
COTTON16_00012_02	1315	0.660	0.654
COTTON16_00056_02	720	0.420	0.431
COTTON16_00922_01	949	0.000	0.007
COTTON16_01121_02	632	0.143	0.017
COTTON16_01189_01	1178	0.172	0.020
COTTON16_01391_01	705	0.818	0.939
COTTON16_01704_01	1415	0.616	0.480
COTTON16_06427_01	504	0.458	0.615
COTTON16_09095_01	1294	0.530	0.579
COTTON16_15666_01	268	0.465	0.309
COTTON16_19029_01	267	0.612	0.489
COTTON16_19620_01	376	0.752	0.812
COTTON16_22170_01	513	0.467	0.266
COTTON16_26306_01	942	0.491	0.449
COTTON16_32946_01	1145	0.487	0.567
COTTON16_34102_01	940	0.635	0.625
COTTON16_34627_01	1149	0.672	0.554

Supplementary Figure 1 b.

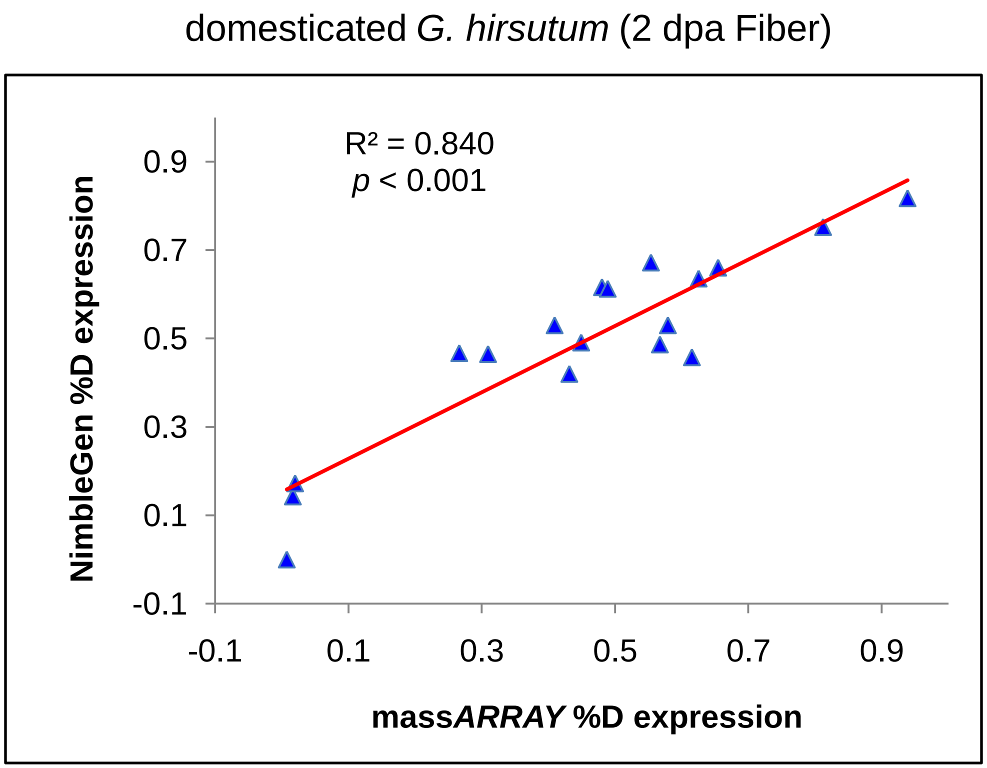

Supplement: Additional file 6 — Validation of NimbleGen microarray values by mass ARRAY-based homoeolog-specific expression measurements. (a) List of 18 homoeologous genes analyzed for validation of NimbleGen microarray values by mass ARRAY-based homoeolog-specific expression measurements. (b) Correlation between NimbleGen microarray data and mass ARRAY-based homoeolog-specific measurements. The relative expression of D-homoeolog detected at 2 dpa based on NimbleGen microarray signal information and plotted on the y-axis. The x-axis is the proportion of D-homoeolog that is observed based on mass ARRAY measurements. The best-fit trend line with R2 correlation and p-value is shown on the graph for early stage of fiber development. The data provided represents the validation of homoeologous gene expression in the developing fibers from wild and domesticated accessions. [file 1471-2164-10-378-S6.pdf]
